# Draft genome of a *Bacillus subtilis* ICS isolated from an okra (*Abelmoschus esculentus*) extract exhibited genes related to plant growth-promoting potential

**DOI:** 10.1128/mra.00736-24

**Published:** 2024-12-13

**Authors:** Paul Christian T. Gloria, Ma. Jhanessa A. Luague, Maria Auxilia T. Siringan, Vina B. Argayosa

**Affiliations:** 1 Natural Sciences Research Institute, University of the Philippines, Diliman, Quezon City, Philippines; SUNY College of Environmental Science and Forestry, Syracuse, New York, USA

**Keywords:** *Bacillus subtilis*, genomics, whole-genome sequencing, plant growth-promoting bacteria, draft genome

## Abstract

Here, we report the draft genome of *Bacillus subtilis* ICS isolated from the aqueous extract of *Abelmoschus esculentus*. The isolate has a genome size of 4.15 Mb, 43.07% GC, 99.8% BUSCO completeness, and 91.15% CheckM completeness. The genome exhibited genes related to plant growth promotion and secondary metabolite production.

## ANNOUNCEMENT

Plant growth-promoting bacteria (PGPB) enhance plant health through various mechanisms, such as systemic resistance, antibiosis, and competitive exclusion (1). One known PGPB is *Bacillus* spp. with beneficial traits for plant and soil health (2). *Bacillus subtilis* is a known rhizosphere-associated bacteria (3) that protect plants from oxidative stress (4) and reduce nitrogen emissions (5).

In this report, we utilized *Bacillus* sp. from the aqueous fraction of *Abelmoschus esculentus* extract submitted for antimicrobial analysis (agar well diffusion) to the Microbiological Research and Services Laboratory. The submitted extract was realized to be unsterile since the sterility control (distilled water) and negative control (Nutrient Broth) did not display apparent contamination upon assay. Thus, we sought to isolate the extract-derived bacteria through serial dilution of the extract and spread plating of the aqueous fraction in Nutrient Agar at 35°C for 18 hours with several streak-plating afterwards. The recovered *Bacillus* sp. ICS was cultured in the same conditions for purification, maintenance, and DNA extraction. DNA was extracted from fresh cultures using the GF-1 Nucleic Acid Extraction Kit (Vivantis Technologies, Malaysia) following the manufacturer’s protocol. Extracted DNA was sent to Macrogen (South Korea) for sequencing using an Illumina NovaSeq 6000 platform with 150 pair-ended settings from TruSeq Nano DNA-prepared libraries. Raw reads were quality-trimmed to Phred30 using Trimmomatic version 0.36 (6). Assembly using Unicycler version 0.5 (7), utilizing three different bridging modes, was evaluated using QUAST version 5.2.0 (8). Only the best assembly is reported here. Afterwards, the completeness and contamination of the genome were checked using BUSCO version 5.5 (9) and CheckM version 1.2 (10). GTDB-Tk version 1.7.0 (11) was used to establish taxonomic identity. General annotation was done using RAST version 1.073 (12) and Prokka version 1.14.5 (13) under default settings. antiSMASH 7.1.0 (14) was used to detect secondary metabolite biosynthetic gene clusters (smBGCs) with a detection strictness of relaxed and all extra features turned on.

Sequencing yielded 1,302,291,346 raw reads. The best assembly was generated using Unicycler’s normal bridging mode, where the genome recovered had a length of 4,150,469 bp, 43.07% GC, *N*_50_ of 55,889, and *L*_50_ of 28, with the largest contig at 198,741 nucleotides and a total of 178 contigs with lengths greater than or equal to 200 bp. Genome coverage was 100× with 99.8% BUSCO completeness, 91.15% CheckM completeness, and 0% contamination. GTDB-Tk assigned the genome to *Bacillus subtilis* with a 95% fastANI reference radius to the reference genome GCF_000009045.1. RAST and Prokka predicted 4,687 and 4,477 genes, respectively. Annotation revealed genes related to nitrogen, phosphate, and potassium metabolism and siderophore production. These genes suggest the potential plant growth-promoting capabilities of the isolate. antiSMASH predicted 14 smBGCs. Out of 14, four encode non-ribosomal peptide synthase, four polyketide synthases, two terpene clusters, and other types. Most of the predicted smBGCs (9 out of 14) have a similarity percentage lower than 40%, suggesting high novelty. This result further underscores the possibility of *Bacillus subtilis* ICS in remodeling the rhizosphere microbiome to create a more fertile plant microenvironment. RAST revealed only one virulence gene, *cvfB*, and 17 antibiotic resistance genes with vancomycin cassettes as most prevalent.

## Data Availability

This genome project has been registered at NCBI under accession number PRJNA1123903. Raw reads were deposited at the NCBI Sequence Read Archive (SRA) under the accession number SRR29409878. The genome sequence has been deposited at GenBank under the accession number JBELOF000000000 with the assembly accessible through GCA_040270045.1. Other annotations have also been uploaded to Zenodo (10.5281/zenodo.13864689).
